# Gene Panel of Persister Cells as a Prognostic Indicator for Tumor Repopulation After Radiation

**DOI:** 10.3389/fonc.2020.607727

**Published:** 2020-11-20

**Authors:** Yucui Zhao, Yanwei Song, Ruyi Zhao, Minghui Zhao, Qian Huang

**Affiliations:** Cancer Center, Shanghai General Hospital, Shanghai Jiao Tong University School of Medicine, Shanghai, China

**Keywords:** ****treatment response, persister cells, tumor repopulation, radiotherapy, prognostic index

## Abstract

Tumor repopulation during cycles of radiotherapy limits the radio-response in ensuing cycles and causes failure of treatment. It is thus of vital importance to unveil the mechanisms underlying tumor repopulating cells. Increasing evidence suggests that a subpopulation of drug-tolerant persister cancer cells (DTPs) could survive the cytotoxic treatment and resume to propagate. Whether these persister cells contribute to development of radio-resistance remains elusive. Based on the genetic profiling of DTPs by integrating datasets from Gene Expression Omnibus database, this study aimed to provide novel insights into tumor-repopulation mediated radio-resistance and identify predictive biomarkers for radio-response in clinic. A prognostic risk index, grounded on four persister genes (LYNX1, SYNPO, GADD45B, and PDLIM1), was constructed in non-small-cell lung cancer patients from The Cancer Genome Atlas Program (TCGA) using stepwise Cox regression analysis. Weighted gene co-expression network analysis further confirmed the interaction among persister-gene based risk score, radio-response and overall survival time. In addition, the predictive role of risk index was validated *in vitro* and in other types of TCGA patients. Gene set enrichment analysis was performed to decipher the possible biological signaling, which indicated that two forces behind persister cells, stress response and survival adaptation, might fuel the tumor repopulation after radiation. Targeting these persister cells may represent a new prognostic and therapeutic approach to enhance radio-response and prevent radio-resistance induced by tumor repopulation.

## Introduction

Radiotherapy constitutes the first line of cancer treatment in over 60% of patients with local advanced solid malignancies (1). Generally, several cycles of radiation application are needed to exert the maximum tumoricidal effect with minimum damage to normal tissue. Although these cytotoxic treatments bring about initial effectiveness in eliminating tumor masses, tumor recurrence inevitably occurs even after several cycles of treatment, which accounts for 90% of clinical death (2). Tumor repopulation, describing the revival and re-propagation of residual living cancer cells between treatment intervals, has been regarded as a major cause of treatment refractory. Hence, understanding the mechanism underlying these repopulating cells is of top priority to overcome cancer resistance.

Recent studies showed that there are always a small subpopulation of cancer cells surviving through the cytotoxic treatments (3), which is reminiscent of a drug-tolerant state of bacteria in response to antibiotics (4). Although the replicating antibiotic-sensitive bacteria are markedly killed, non-growing or slow-cycling bacteria bearing greater tolerance are able to resume growth and reestablish a sensitive population upon drug withdrawal. Similar phenomenon of drug-tolerant persisters (DTP) had later been described across a wide range of cancer types, such as non-small-cell lung cancer (NSCLC) (5), melanoma, breast cancer, and ovarian cancer (6). It was estimated that 20% of DTPs could eventually revert to proliferative state and develop to “drug-tolerant expanded persisters” (DTEPs) (5, 7). The slow-cycling state of DTP was recognized as the precondition for further transcriptional reprogramming on the way to DTEPs (8, 9). Compared with parental cells, DTPs exhibited an increased dependency on main anti-apoptotic effectors, BCL-2, and BCL-XL, which might be regulated through ER stress signaling (10, 11). In addition, these DTPs highly expressed some cancer stemness markers, like CD133, which probably play an essential role in the survival and proliferation through cytotoxic drug treatment (5, 9, 12).

Based on the fact that DTPs serve as founders in drug-resistance from lower microorganism to mammalian (4, 13), it seems that DTPs represent a more primitive and evolutionally conserved phenotype in response to stress. However, persister cells are originally discovered in condition of drug treatment while there are rare researches concerning this phenomenon in radiation, we thus wonder whether persister cells contribute to tumor repopulation after radiotherapy in the same manner. In this study, we aimed to explore the role of persister cells in tumor repopulation following radiotherapy so as to identify certain persister-gene signatures for monitoring the radio-resistance and predicting clinical outcome. Besides, to make the most of the potential clinical translational value of our work, we analyzed the potential biological pathways, which might provide new avenues to improve the benefit of radiotherapy.

## Materials and Methods

### Clinical Samples and Data Acquisition

In this study, we searched “NSCLC” and “persister cells” as the keywords in the Gene Expression Omnibus (GEO) database (http://www.ncbi.nlm.nih.gov/geo/). Datasets of GSE153183 and GSE114647 with paired parental and tyrosine kinase inhibitor (TKI)-induced persister cells were downloaded using the R package “GEOquery”.

We further enrolled radiation-treated patients from The Cancer Genome Atlas Program (TCGA) database, in which NSCLC cohorts (lung adenocarcinoma and lung squamous cell carcinoma, LUAD and LUSC, n = 20) were set as the training set, while rectum adenocarcinoma (READ, n = 15), cervical squamous cell carcinoma and endocervical adenocarcinoma (CESC, n = 101) and esophageal carcinoma (ESCA, n = 41) cohorts were used as the validating sets. The mRNA expression (Illumina HiSeq 2000) of these TCGA samples with clinical annotations containing radiotherapy details and overall survival (OS) information was acquired with the R package “TCGAbiolinks”. According to the clinical response status from TCGA datasets, NSCLC patients were allowed to be classified as “sensitive” to radiation, including complete response (CR) or partial response (PR), as well as “resistance” to radiation with progressive or recurrent diseases.

### Analysis of Differentially Expressed Genes

Background correction, log2 transformation, and quantile normalization were firstly implemented for each GEO sets. The “limma” package was then performed to screen the genetic profiling of DTP. DEGs between parental cells and persister cells were obtained using |log2FC|>1 and p value <0.05 as cut-off values. For pathway and function enrichment analysis of DEGs, “Metascape”, the web-based portal, was employed to annotate and integrate the results (http://metascape.org/) (12).

### Construction and Analysis of Prognostic Signature

Based on 76 upregulated DEGs of DTP, we used univariate Cox regression analysis to identify significant prognostic genes (p < 0.05) related with OS of radiation-treated NSCLS patients. Then a multivariate Cox proportional hazards regression model was constructed using the following formula,

$$Risk\;score=\Sigma_{i=1}^{n}(Coefi\times xi)$$

where *Coefi* represents the coefficient and the *xi* is the mRNA expression of highly related genes. The patients were divided into high- and low-risk groups according to the median risk score. To verify the efficiency of the persister-gene based risk score, receiver operating character (ROC) curves were further performed and the area under the curve (AUC) values were compared.

To reduce the effects of confounding clinical factors, we further combined the clinical characteristics of TCGA patients, including gender (male/female), age (whether or not superior to 60), T, N stage, race (white/others), to judge whether the risk index is an independent prognostic factor. Multivariate Cox regression was analyzed and the value of *p* < 0.05 was considered as statistical significance. The nomogram model based on these clinical information and risk score was constructed using R package “rms” (13, 14).

### Predicting the Clinical Radio-Therapeutic Response

To assess the efficiency of the persister-gene panel in predicting radio-resistance, we used R package “glmnet” to build Logistic regression models in different TCGA datasets. AUC values of different persister-gene combinations were calculated and compared to select the best signature for each cancer type.

### Weighted Gene Co-Expression Network Analysis

WGCNA is a biological method to systematically analyze gene expression patterns in multiple samples, which cluster genes with close interconnection into gene modules, thus enabling to explore association of gene modules with clinical traits (15). Using “WGCNA” package, we analyzed TCGA-NSCLC samples to identify the most representative transcriptional modules with the worst clinical outcomes. Only the top 10,000 genes with the highest variance were selected as input data. The function of *GoodSamplesGenes* was used to identify outliers and no outlier sample was detected to exclude. An appropriate soft threshold power was subsequently chosen to develop the scale-free co-expression network. The topological overlap measure (TOM) was evaluated based on the constructed adjacency matrix and the genes were then clustered in different dendrograms according to the corresponding dissimilarity (1-TOM). Dynamic tree cut was conducted and merged dynamic clusters with higher similarity were further accomplished. The correlation between module and clinical traits was calculated and the persister-genes involved modules were then selected to analyze.

### Gene Set Enrichment Analysis

GSEA software (version 4.1.0, http://software.broadinstitute.org/gsea/index.jsp) was applied in the two groups, “high *vs* low risk” and “radio-resistant *vs* sensitive”, with reference to enrich hallmark gene sets from the Molecular Signatures Database (MSigDB). For this analysis, the most highly enriched signal pathways were selected based on the value of normalized enrichment score (NES); nominal p-value <0.5 was set as the cut-off value for GSEA results.

### Cell Culture and Irradiation

Human NSCLC cell lines used in this study (A549 and H460) were purchased from the Chinese Academy of Science Cell Bank (Shanghai, China). A549 and H460 were cultured in RPMI-1640 medium (Life Technologies, USA) supplemented with 10% fetal bovine serum (FBS) and 1% penicillin/streptomycin (both from Gibco; Thermo Fisher Scientific, USA) at 37°C with 5% CO_2_. For radiation assays, the cells were irradiated or sham-irradiated by an X-ray generator (Faxitron, USA) in our hospital with a dose rate of 3.0 Gy/min.

### Clonogenic Formation Assay

A459 and H460 were seeded in 6-well plates in triplicate (100, 200, 1,000, 2,500, 10,000, 20,000 cells per well) and incubated for 24 h before irradiation. The next day, cells were exposed to various doses of radiation (0, 2, 4, 6, 8, 10 Gy, respectively). The plates were fixed with 4% paraformaldehyde (Sangon Biotech, China) and stained with crystal violet (Beyotime Biotechnology, China) after 14 days. The number of colonies containing more than 50 cells were counted and surviving fraction was calculated using linear-quadratic model following the published protocol (16).

### Quantitative Real-Time Polymerase Chain Reaction

Total mRNA was prepared using RNA-extracting reagent RNAiso Plus and reverse transcribed with the PrimeScript™ RT Master Mix Kit (both from Takara, Japan). Q-PCR was performed with TB Green^®^ Premix Ex Taq™ Kit (Takara, Japan) according to the manufacturer. Relative gene expression was analyzed based on the equation 2^−△△CT^ and results were obtained at three independent experiments. The following primers were used:

LYNX1 5′-CCACGCGCACCTACTACAC-3′ (Forward), 5′-TGCAGAGGTCGTACTGGCA-3′ (Reversed)
GADD45B 5′-GCCCTGCAAATCCACTTCAC-3′ (Forward), 5′- GTGTGAGGGTTCGTGACCA-3′ (Reversed)
SYNPO 5′-GGCTGAGTCATCTGTGGAGG-3′ (Forward), 5′-CGGCCCAACGTCTGCTA-3′ (Reversed)
PDLIM1 5′-CCCAGCAGATAGACCTCCAG-3′ (Forward), 5′-TCTGAGCTTCCAAGTGTGTCATA-3′ (Reversed)

### Statistics Analysis

Data were presented as mean ± SD (standard deviation). Statistical significance was defined as follows: n.s, not significant; *P ≤ 0.05; **P ≤ 0.01; ***P ≤ 0.001. Two tailed student’s *t*-test or two-way ANOVA test was adopted for mean comparisons using GraphPad prism 5 software. All statistical tests were two-sided and analysis mentioned above were implemented in R/Bioconductor software (version 4.0.2, http://www.r-project.org). R packages used in this study include: “GEOquery”, “TCGAbiolinks”, “clusterProfiler”, “org.Hs.eg.db”, “limma”, “stringr”, “ggplot2”, “pheatmap”, “dplyr”, “glmnet”, “survminer”, “survival”, “rms”, “foreign”, “survivalROC”, “ROCR”, “WGCNA”.

## Results

### Genetic Profiling of DTP in NSCLC

**Figure 1:**
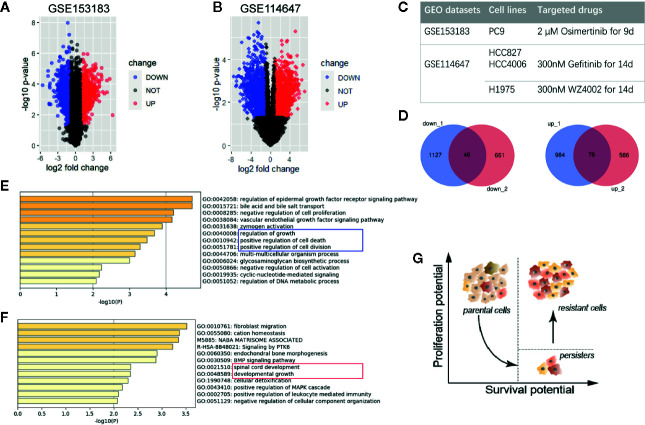
Differentially expressed genes of drug-tolerant persister cells (DTP) in NSCLC. **(A, B)** Volcano plots of GSE153183 and GSE114647. Blue/red plots indicate the down-regulated/upregulated DEGs with the cut-off criteria: |log2FC|>1 and *p* value <0.05. **(C)** Experimental design of two GEO datasets involved in this study. Approaches to construct DTP modules including NSCLC cell lines and target drug treatment are presented. **(D)** Overlapped down-regulated and upregulated DEGs as Venn diagrams shown. **(E, F)** Functional enrichment analysis of down-regulated and upregulated DEGs, colored by *p*-value. **(G)** Schematic diagram of phenotypical transition in cytotoxic drug therapy, representing the evolution of proliferation and survival potential from parental cells to DTPs and the eventual tumor repopulation (resistant cells). DEG, differentially expressed gene; DTP, drug-tolerant persister cell; NSCLC, non-small-cell lung cancer.

To reveal the character of persister cells, GSE153183 and GSE114647 datasets were analyzed (**Figure 1**). Here, data from TKI-treated NSCLC cell lines were preferentially focused on since the concept of DTP was mostly studied *in vitro* in epidermal growth factor receptor (EGFR)-mutant NSCLC (**Figure 1C**). Based on the cut-off with |log2FC|>1 and *p* value <0.05, 2,233 DEGs of persister cells compared with parental cells were recognized in GSE153183 dataset, comprising 1,060 upregulated genes and 1,173 down-regulated genes (**Figure 1A**). Expression profile of GSE114647 displayed 1,369 DEGs, in which 707 genes were upregulated and 662 genes were down-regulated (**Figure 1B**).

Venn plots were used to integrate these DEGs, and we obtained 46 down-regulated genes and 76 upregulated genes as the overlapping gene profile of persister (**Figure 1D**). Gene Ontology analysis (GO) using Metascape tools showed that down-regulated genes were predominantly enriched in cell growth, cell division, cell death and biochemical metabolisms, in accord with the dormant state that was already reported in previous researches (9, 17) (**Figure 1E**; **Figure S1**). On the other hand, upregulated genes were mainly involved in the following GO terms: 1) fibroblast migration, endochondral bone morphogenesis, and BMP signaling pathway; 2) spinal cord development and developmental growth; 3) cellular detoxification (**Figure 1F**; **Figure S1**). Consistent with the model posit by Chisholm et al., the population of persister cells are able to weather the storm of treatment and possess lower level of proliferation potential than parental cells (6). Despite in a long-term of latency, these persister with higher level of survival potential are believed to propagate into DTEP and result in the final recurrence (**Figure 1G**).

### Identification of a Persister Gene Panel for Predicting Clinical Outcomes of Radiation in NSCLC

**Figure 2:**
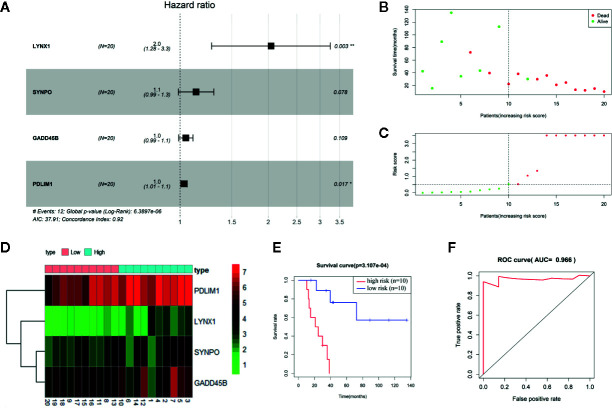
Construction of persister-gene based prognostic signature in NSCLC. **(A)** A radiotherapy prognostic signature containing four persister genes was developed using stepwise regression analysis. Risk ratio (HR), 95% confidence interval, and coefficient in forest map were calculated by multivariate Cox regression analysis. **(B)** Distribution of survival time in enrolled NSCLC patients who received radiotherapy (n = 20). **(C)** Patients were separated into high- and low-risk groups based on the median of risk scores. **(D)** Gene expression matrix of four key persister genes in high- and low-risk groups. Green, red, and black respectively define a lower expression level, a higher expression level, and no expression difference. **(E, F)** Survival curve and ROC curve validation of the prognostic efficiency of the risk score. AUC, area under curve; HR, hazard ratio; ROC, receiver operating characteristic.

To establish the persister gene index for prognosis in NSCLC patients treated with radiation, we performed univariate and multivariate Cox proportional hazards regression analysis in NSCLC patients (n = 20) from TCGA database (18, 19). As the univariate Cox analysis listed in **Table S3**, among the above-mentioned 76 upregulated genes of persister cells, six genes were collected with their significant association with OS of patients (*p* < 0.05). All these survival-related genes (LYNX1, GADD45B, P2RX6, PDLIM1, SYNPO, and MEIS3) were identified as “high-risk” factors, with hazard ratios (HR) superior to 1. To optimize the persister-based gene panel, multivariate Cox regression model was conducted in the range of these six genes, leaving four genes to construct the prognostic gene model: Risk score = 0.714 × LYNX1 + 0.126 × SYNPO + 0.046 × GADD45B + 0.031 × PDLIM1 (**Figure 2A**). As **Figure 2B** illustrated, distribution of patients’ risk score was closely linked to their survival status, confirming good performance of this prognostic model. According to the risk formula, all patients were divided into two groups: high-risk (n = 10, risk scores were from 0.539 to 518.236) and low-risk (n = 10, risk scores were from 0.002 to 0.526) (**Figure 2C**). The heat map of **Figure 2D** curated expression level of the four key genes and their relationship with risk groups. Grounded on the two groups, we further drew the survival curve and 3 year-ROC curve for enrolled 20 NSCLC patients. As shown in **Figure 2E**, higher risk score was tightly correlated with worse prognosis. Similarly, AUC of 3 year-ROC curve was 0.966, indicating that using the persister gene panel to predict prognosis of radiotherapy is highly reliable.

After Cox analysis combined with clinical features, risk score was still proved as an independent prognostic factor (*p* = 0.020) (**Figure S2**). The nomogram for evaluating 1-, 3-, 5-year OS was further established based on clinical characteristics and risk score (**Figure S2**). Because NSCLC patients enrolled were all at stage M0, the nomogram model contains six factors: sex, age, T, N stage, race, and risk score. The C index was 0.927, indicating a good predictive ability.

### The Persister Gene Panel as an Indicator for Radio-Resistance and Tumor Repopulation

**Figure 3:**
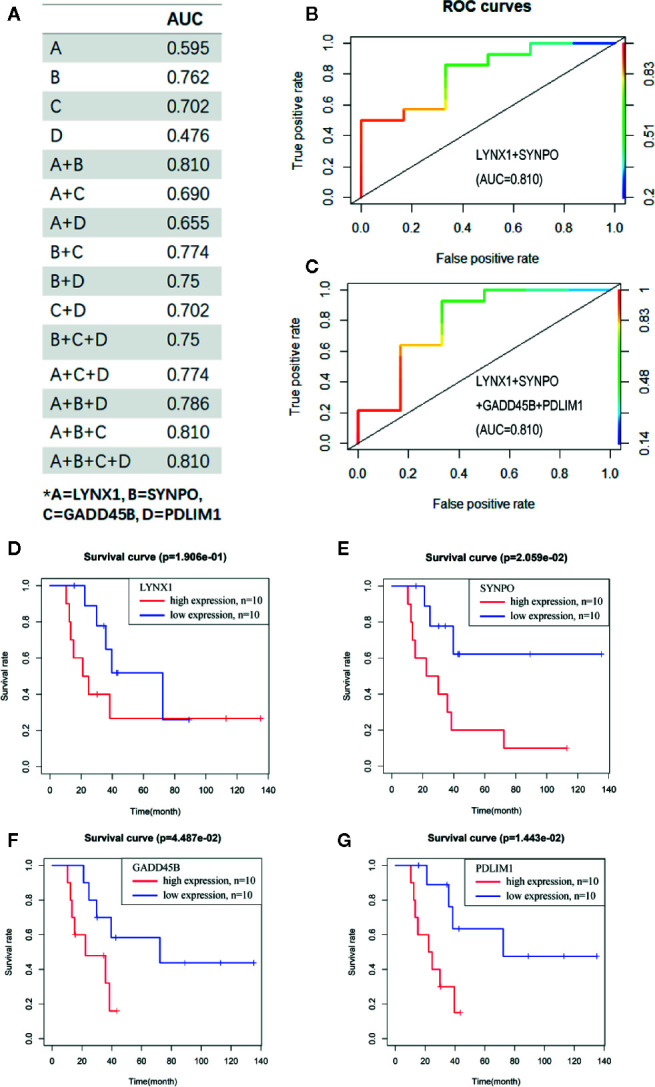
The persister-gene panel acts as an indicator of poor clinical outcome of radiotherapy in NSCLC. **(A)** AUC values of the combined persister genes in ROCs for estimating them as indicators of radio-resistance. ROCs were constructed with Logistic regression model. **(B, C)**. The best AUC values were shown as the combination of LYNX1 and SYNPO, as well as the four-gene group. **(D–G)**. Survival curve of LYNX1, SYNPO, GADD45B, and PDLIM1, according to the Kaplan–Meier analysis. Red/blue lines indicate high/low expression of the corresponding genes.

To evaluate the potential sensitivity and specificity of the persister-based gene panel in tumor response judging following radiation, multivariate logistic regression analysis was used to calculate concomitant administration of genes in predicting treatment response. Compared with AUCs of single biomarker candidates (from 0.476 of PDLIM1 to 0.762 of SYNPO), the AUC values of two or more gene groups were generally increasing (20) (**Figure 3A**). Among all combination, the AUC value of LYNX1 combined with SYNPO was equal to the union of four genes and reached the highest, 0.810 (**Figures 3B, C**). In addition, survival curves of single persister genes demonstrated a coincident trend: higher expression of persister genes indicated worse clinic outcome (*p* = 0.191, 0.021, 0.045 and 0.014 for LYNX1, SYNPO, GADD45B, and PDLIM1, respectively) (**Figures 3D–G**). All these data substantiate the hypothesis that persister gene panel may be employed as a reliable indicator to monitor tumor resistance and tumor repopulation following radiotherapy in NSCLC.

### Correlation of Persister Gene Panel and Clinical Traits of Radiotherapy Confirmed by WGCNA

**Figure 4:**
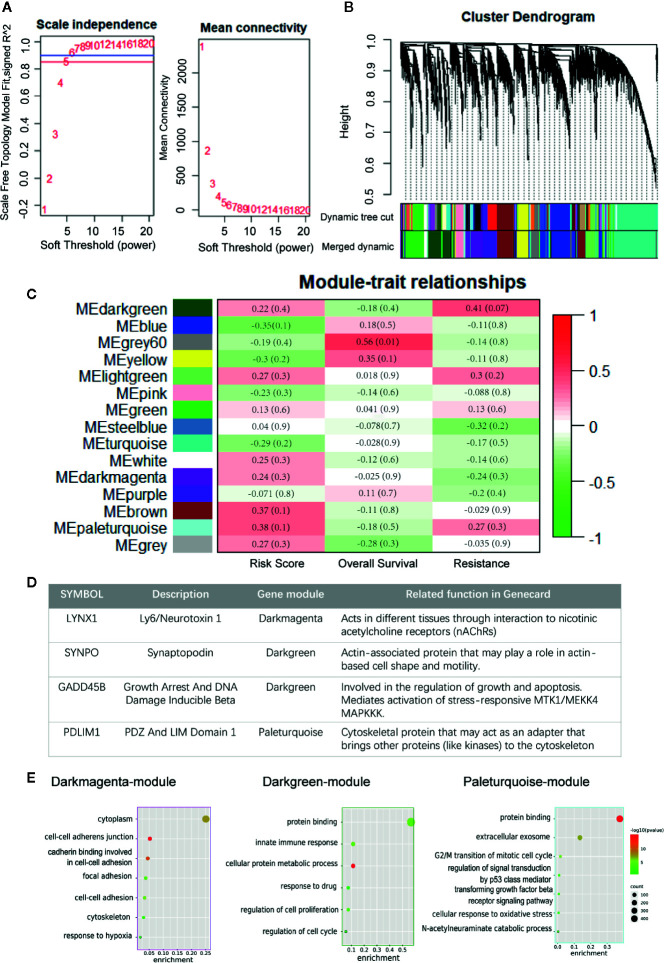
Construction of weighted co-expression network. **(A)** Scale independence and mean connectivity for various soft thresholds (powers). **(B)** Hierarchical clustering tree of the top 10,000 genes with dissimilarity. Gene modules were shown with dynamic tree cut and further merged dynamic. **(C)** Heatmap of module–traits relationships. Each column represents a clinical trait and rows correspond to module. Correlation and *p*-value levels are labeled in each bracket. **(D)** Description of four persister-genes and their ownerships to different gene modules. **(E)** Dot plots of the GO biological processes enrichment in dark magenta, dark green and paleturquoise module, which contains LYNX1, SYNPO, and GADD45B, PDLIM1, respectively. The dot size and color represent the gene count and enrichment level (*p*-value), respectively. GO, Gene Oncology.

In this study, we performed WGCNA on the top 10,000 expressed genes of TCGA NSCLC patients to further ascertain the interrelation among persister gene panel, risk score of prognosis and radio-resistance. The power *β* = 6 (scale free topology fitting index R^2^ >0.85) was selected as the soft-threshold value to construct scale-free network (**Figure 4A**). As shown in **Figure 4B**, 15 genes modules with gene numbers greater than 50 were identified after merged dynamic tree cutting (**Figure 4B**). In relating these gene modules to clinical information, we found that gene modules with higher predicted risk score held higher possibility of resistance to radiation and lower overall survival time, which served as a strong cross-validation for the predictive role of persister genes-based risk formula in radiotherapy (**Figure 4C**).

Afterwards, we found that the four persister genes belonged to three gene modules with relatively high risk score correlation index (R^2^): LYNX1 in dark magenta module with 0.236 (*p* = 0.317), SYNPO and GADD45B in dark green module with 0.220 (*p* = 0.351), PDLIM1 in paleturquoise module with 0.382 (*p* = 0.096) (**Figure 4D** and **Tables S8, S9**). Except the low correlation of resistance in darkmagenta module (R^2^ = −0.242, *p* = 0.302), dark green and paleturquoise modules bore relatively high correlativity with radio-resistance (R^2^ = 0.414, *p*= 0.070 and R^2^ = 0.269, *p*= 0.253) and negative outcome in OS (R^2^ = −0.183, *p* = 0.0.439 and R^2^ = −0.163, *p* = 491). These data unbiasedly confirmed the tight correlation between expression of the persister gene panel and clinical outcome of radiotherapy. Furthermore, the three gene modules were chosen for GO analysis and we found that their functional network were more involved in cell adhesion, cell proliferation, cell cycle pathway and other stress signaling (**Figure 4E**).

### Associated Biological Pathways by GSEA Analysis

**Figure 5:**
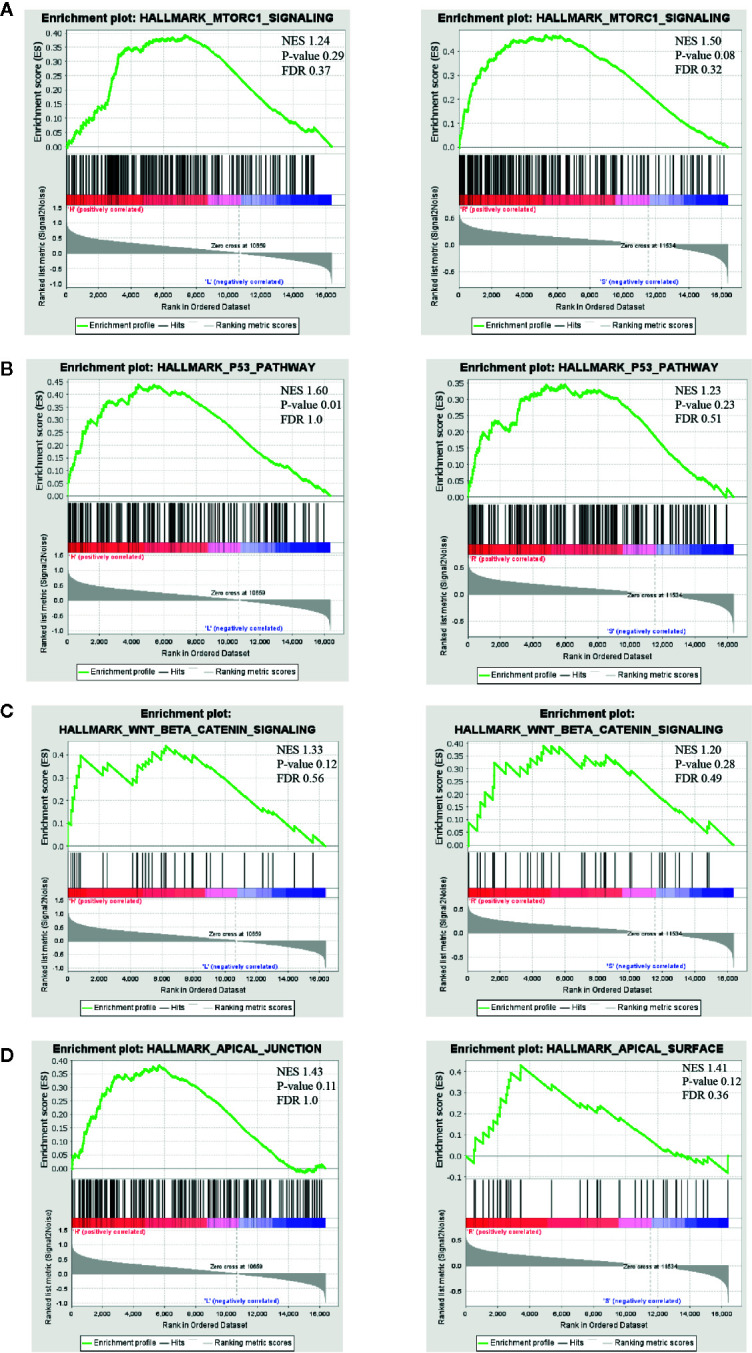
Possible biological pathways identified by gene set enrichment analysis (GSEA). The highly enriched signal pathways were selected based on the cut-off value: normalized enrichment score (NES) >1. The common pathways between “high- *vs* low-risk” (left panel) and “radio-resistant *vs* sensitive” groups (right panel) are displayed in **(A–D)**. NES, nominal *p*-value and FDR are shown in each plot (upper right). mTORC1, mammalian target of rapamycin complex 1; NES, normalized enrichment score.

Understanding the resistant mechanisms behind persister cells is of importance for further hitting these potential targets. Two groups, “high *vs* low risk” and “radio-resistant *vs* sensitive”, were analyzed with GSEA software. Compared to risk-low groups, expression of risk-high patients was more relevant to “MTORc1 signaling”, “P53 pathway”, “wnt-beta catenin signaling” and “apical junction” (**Figures 5A–D**, left panels and **Table S5**). Similar pathways were significantly enriched in radio-resistant patients (**Figures 5A–D**, right panels and **Table S6**). Combined with GO enrichments in WGCNA, we deducted that persister cells maintained a dynamic persistence, which is characterized by a balance between stress response (P53 and mTOR signaling regulated) and survival adaptation (wnt-beta catenin and apical junction related).

### Validation That Persister Gene Panel Is Related to Tumor Repopulation After Radiation *In Vitro* and in Other Cancer Types

**Figure 6:**
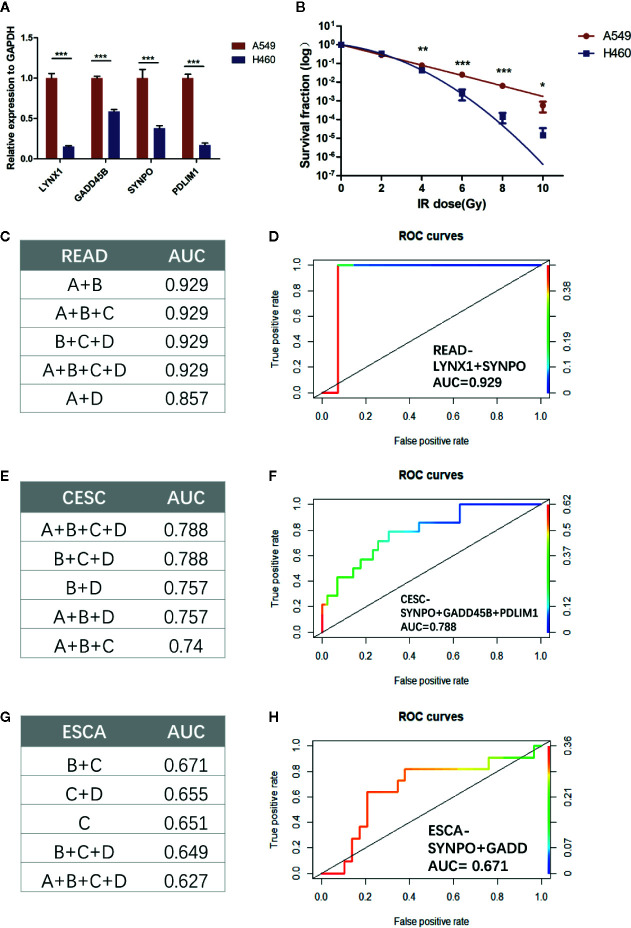
Validation the role of persister-gene panel in predicting radio-resistance in NSCLC *in vitro* and in other cancer types. **(A)** In order to compare the baseline expression of persister-gene panel in two NSCLC cell lines (A549 and H460), relative expression of associated genes’ mRNA was assessed by qRT-PCR conducted in three independent experiments, each done in triplicate (normalized against GAPDH). Data represent mean values ± standard deviation. ***p ≤ 0.001 **(B)** Survival curves show the survival fraction of A549 and H460 after irradiation performed by clonogenic formation assay in triplicate. Data represent mean values ± standard deviation. P-values were obtained by two-way ANOVA test; *p ≤ 0.05; **p ≤ 0.01; ***p ≤ 0.001 **(C, E, G)** The top five AUC values of the combined persister genes for estimating them as indicators of radio-resistance in READ (n = 15), CESC (n = 101), and ESCA (n = 41), respectively. **(D, F, H)** The least number of gene-panel combination with the highest AUC value were exhibited. CESC, cervical squamous cell carcinoma and endocervical adenocarcinoma; ESCA, esophageal carcinoma; READ, rectum adenocarcinoma.

Considering the limited number of NSCLC patients enrolled, we firstly applied our persister-based risk formula *in vitro*. As **Figure 6A** shown, NSCLC cell line A549 displayed higher transcriptional expression of the four persister genes relative to H460. It turned out that the survival fraction of A549 was significantly higher than H460, which was consistent with our expectation (**Figures 6B**, **S3**). Furthermore, we expanded the persister-gene panel in other cancer types which were frequently treated with radiotherapy, including READ (n = 15), CESC (n = 101), and ESCA (n = 41) from TCGA database. To comprehensively assess clinical predictive role of the persister gene panel in radiotherapy, logistic regression analysis were performed and the top five AUCs for each cancer types were listed in **Figure 6**. Results revealed that the persister gene panel had the highest predictive ability in radio-resistant outcome of READ patients (AUC = 0.929) with the combination of LYNX1 and SYNPO (**Figures 6C, D**). The four gene panel exhibited a good performance in radio-response prediction for CESC patients (AUC = 0.788) (**Figures 6E, F**). As for ESCA patients who received radiotherapy, the panel of SYNPO and GADD45B displayed the best specificity and sensitivity in resistance forecast (AUC = 0.671) (**Figures 6G, H**).

## Discussion

Cytotoxic therapies brutally kill cancer cells; however, the progressive emergence of resistance remains a critical obstacle for achievement of cures. Tumor repopulation of residual cells has been recognized as a priming factor in tumor resistance (21). Firstly found in fractioned radiation, tumor repopulation was also observed in chemotherapy, especially after multiple doses (22, 23). Although it was acknowledged that cancer stem cells (CSCs) largely constituted the repopulating cells, lack of unique or invariable biological properties makes the concept of CSC somewhat controversial. Accumulating studies have demonstrated that a small subpopulation of DTPs could resist the initial onslaught of cytotoxic agents for a long term until further mutations can be evolved (4, 24). Some of these slow-cycling persister cells resumed proliferative potential and eventually repopulated the tumor. However, whether these persister cells are likewise involved in tumor repopulation after radiation is far from being well understood.

To address this question, we firstly turned our eyes on TKI-induced persister cells in NSCLC given the vast number and solid evidence of researches on this model. By integrating high-throughput sequencings of GEO, genetic profiling of DTPs were identified in NSCLC. Based on these upregulated genes in persister cells, we developed a four genes-composed radio-resistance signatures in NSCLC patients from TCGA datasets. The persister-gene panel could not only provide prognostic value for NSCLC, but was also validated in other three types of cancers. To our knowledge, our work creatively demonstrates that there is a significantly positive relation between persister cells and tumor repopulation after radiotherapy. Furthermore, the persister gene panel enables tumor assessment as early as the first cycle of radiotherapy, which might guide the adjustment of treatment schedule and ameliorate tumor repopulation.

In this study, we firstly found 46 down-DEGs and 76 up-DEGs of drug-induced persister cells compared to parental cells (**Figures 1G**, **S1**). Down-regulated pathways reflected a dormancy but drug-tolerant profile of DTP, which might be a response to stress-induced DNA damage (**Figure 1E**). To maintain genomic integrity of normal cells, damaged DNA induces checkpoints (*e.g.* p53-p21)-controlled cell cycle arrest until that DNA repair is achieved or cell death is launched (25). Under this premise, malignant cells might sustain in G0 phase by activating other epigenetics or genetics-dependent quiescence programs (26). It is the long-term slow-cycling state that enables additional mutations for permanent resistance and later repopulation. Furthermore, this latent state protects persister cells not to be detected or eliminated by immune surveillance, which is responsible for tumor progression (27). On the other hand, upregulated pathways are enriched in higher survival potential, including developmental growth, cellular plasticity and detoxification, attributing to regeneration and robust adaptation of DTP (**Figure 1F**). Thereinto, epithelial–mesenchymal transition (EMT) is the best-known mechanism for cell plasticity, encompassing loss of cell-cell junction, increased fibroid morphology, extracellular matrix-associated migration and resistance to apoptosis (28). Increasing data suggested that EMT exerts an essential influence on the emergence of persister cells (29, 30). Overall, this genetic profiling of DTP corresponds to minimal residual disease in clinic, which represents as a transitory stage between initial response and progressive recurrence.

Whether these persister cells with low proliferative but high survival potentials are engaged in tumor repopulation is still largely unknown. Hence, in this study, we focused on the predictive value of drug-elicited persister DEGs in patients treated with radiotherapy. Utilizing the multiple factor Cox regression model in NSCLC patients, a prognostic gene signature based on persister profiling was constructed (**Figures 2** and **3**). Four “high-risk” genes, LYNX1, GADD45B, SYNPO and PDLIM1, were further validated their prognostic ability with radio-resistance in READ, CESC and ESCA patients in different combination (**Figure 6**). As **Figure 4D** summarized, one of the Ly-6 protein family, lynx1, is a negative allosteric modulator of nicotinic acetylcholine receptor (nAChR). Lynx1 is expressed widely in squamous lung cancer, but with a significantly decreased level relative to normal adjacent tissue (31). Knock-down of LYNX1 enhanced the growth of A549 cells; overexpression of LYNX1 conversely resulted in cell cycle arrest in lung cancer (32). A recent study revealed that this growth-suppressing role of LYNX1 was due to activation of several kinases cascades after interaction with *α*7-nACHRs (33). GADD45B, a member of the growth arrest and DNA damage-inducible gene family, is previously regarded as a tumor suppressor gene, which participate in apoptosis, growth arrest and DNA damage repair through TP53 or other means (34). This seems to be conflictive to our results, where high level of GADD45B was a radio-resistance predictive marker. However, the promoting role of GADD45B in tumorigenesis or rapid disease progression was also reported in colorectal carcinoma (35, 36), gastric (37), and ovarian cancer (38). As a stress-response gene, GADD45B might be continuously activated with the accumulation of DNA damage and loss its normal function in the tumor progression. SYNPO encodes the proline-rich, actin-associated protein, synaptopodin, which is enriched in highly dynamic compartments and mostly studied in podocyte of kidney (**Figure 4D**). Ectopic expression of synaptopodin repressed the migration of human breast cancer MDA-MB 231 (39), which is consistent with the lower migration capability of persister cells with a high expression of SYNPO than parental cells. PDZ and LIM domain protein 1 (PDLIM1), is also an EMT-associated cytoskeleton protein (**Figure 4D**). Similarly, elevated expression of PDLIM1 was associated with a reduced invasive ability of colorectal (40) and hepatocellular cancer cells (41), which coincides with the mesenchymal phenotype of therapy-induced persistence.

What we next focused on was the underlying mechanisms that persister cells engaged in the radio-resistance. The interesting findings of GSEA analysis (**Figure 5**) implicated a subtle balance between the brake signaling of proliferation and enhanced ability of survival potential in both “risk-high” and “resistant” groups. As mentioned before, p53 functions as a stress sensor to trigger cell-cycle arrest in response to cytotoxic treatment. Mechanistic target of rapamycin complex 1 (mTORC1) signaling is also an evolutionally conserved stress sensor to couple cell growth with nutrients and energy (42). Activation of MTOR signaling was shown to confer multiple cancer types with resistance to chemo-radiation (42, 43). An elegant work recently using whole-genome RNAi screening identified MTOR as a common orchestrator in stress-induced genomic instability, accelerating the adaptation of cancers in cytotoxic condition and facilitating the cancer resistance to different oncotherapy (44). Moreover, the combination of 5-FU and temsirolimus, an mTOR inhibitor, reduced the chemo-resistance related persister cells in gastric cancer (8). Besides TP53 and MTOR mediated lower proliferation, wnt/beta-catenin signaling was revealed here to be associated with higher survival potential. Briefly, binding of Wnt ligands initiates this canonical pathway, inducing translocation of the nuclear localization of beta-catenin and activating wnt downstream targets to potentiate cell growth, adhesion and survival (45). Deregulated expression of wnt/beta-catenin has been reported in different cancer progression and therapeutic tolerance (46–48). In addition, dysregulation of apical junction is tightly connected with EMT (49), which contributes to the flexibility of cell motility and tissue regeneration.

There are some limitations in current study. For instance, transcriptome analysis cannot cover the overall alteration in chemo-radiation induced persister cells, especially the epigenetics change. Meanwhile, *in vivo* confirmation is necessary to validate the role of persister-gene panel in tumor repopulation after radiation and its generalizability in other cancer types. With the rapid pace of advancement in high-throughput sequencing and novel experimental technologies, higher level and deeper dimension of molecular mechanisms behind the persisters are awaiting to be evaluated.

In conclusion, our research outlined the genetic characteristic of DTP based on GEO datasets and applied it to NSCLC patients from TCGA. The persister gene panel (LYNX1, SYNPO, GADD45B, and PDLIM1) was established and its role to elucidate radio-response and clinical outcome after radiotherapy was subsequently validated across several cancer types. Finally, the downstream pathways behind persister cells were disclosed, potentially providing therapeutically exploitable vulnerabilities of tumor repopulation.

## Data Availability Statement

The datasets presented in this study can be found in online repositories. The names of the repository/repositories and accession number(s) can be found in the article/**Supplementary Material**.

## Author Contributions

YZ and YS made equal contribution to this study. Conceptualization, YZ and QH. Data curation, YZ and YS. Methodology, RZ, MZ, and QH. Project administration, YZ and YS. Writing, YZ. Review and editing: YS, RZ, MZ, and QH. All authors contributed to the article and approved the submitted version.

## Funding

This study was supported by a funding from National Natural Science Foundation of China (No. 81972843 to QH).

## Conflict of Interest

The authors declare that the research was conducted in the absence of any commercial or financial relationships that could be construed as a potential conflict of interest.

## Supplementary Material

Supplementary Figure 1 Chord diagrams of gene ontology analysis of down-regulated **(A)** and upregulated **(B)** DEGs in DTPs.

Supplementary Figure 2 Cox regression analysis of patient clinical information and the nomogram model for predicting abilities of NSCLC patients with 1-, 3-, and 5-year OS.

Supplementary Figure 3 Representative images of clongenic formation assay for comparison of A549 and H460.

Supplementary Table 1 DEGs of datasets GSE153183.

Supplementary Table 2 DEGs of datasets GSE114647.

Supplementary Table 3 Persister genes shown with significance (p < 0.05) selected by univariate COX analysis.

Supplementary Table 4 Risk score of TCGA-NSCLC patients enrolled in this study based on the persister gene expression.

Supplementary Table 5 GSEA reports for “high-risk” patients.

Supplementary Table 6 GSEA reports for “radio-resistant” patients.

Supplementary Table 7 Top 10,000 expressed genes in WGCNA analysis.

Supplementary Table 8 Correlation of module and clinical traits in WGCNA analysis.

Supplementary Table 9
*P*-value of module and clinical traits in WGCNA analysis.
