# Supplementary figures and images for: Gene Panel of Persister Cells as a Prognostic Indicator for Tumor Repopulation After Radiation
Source: Front Oncol. 2020 Nov 20;10:607727. doi: 10.3389/fonc.2020.607727 (PMC7714959; doi:10.3389/fonc.2020.607727)

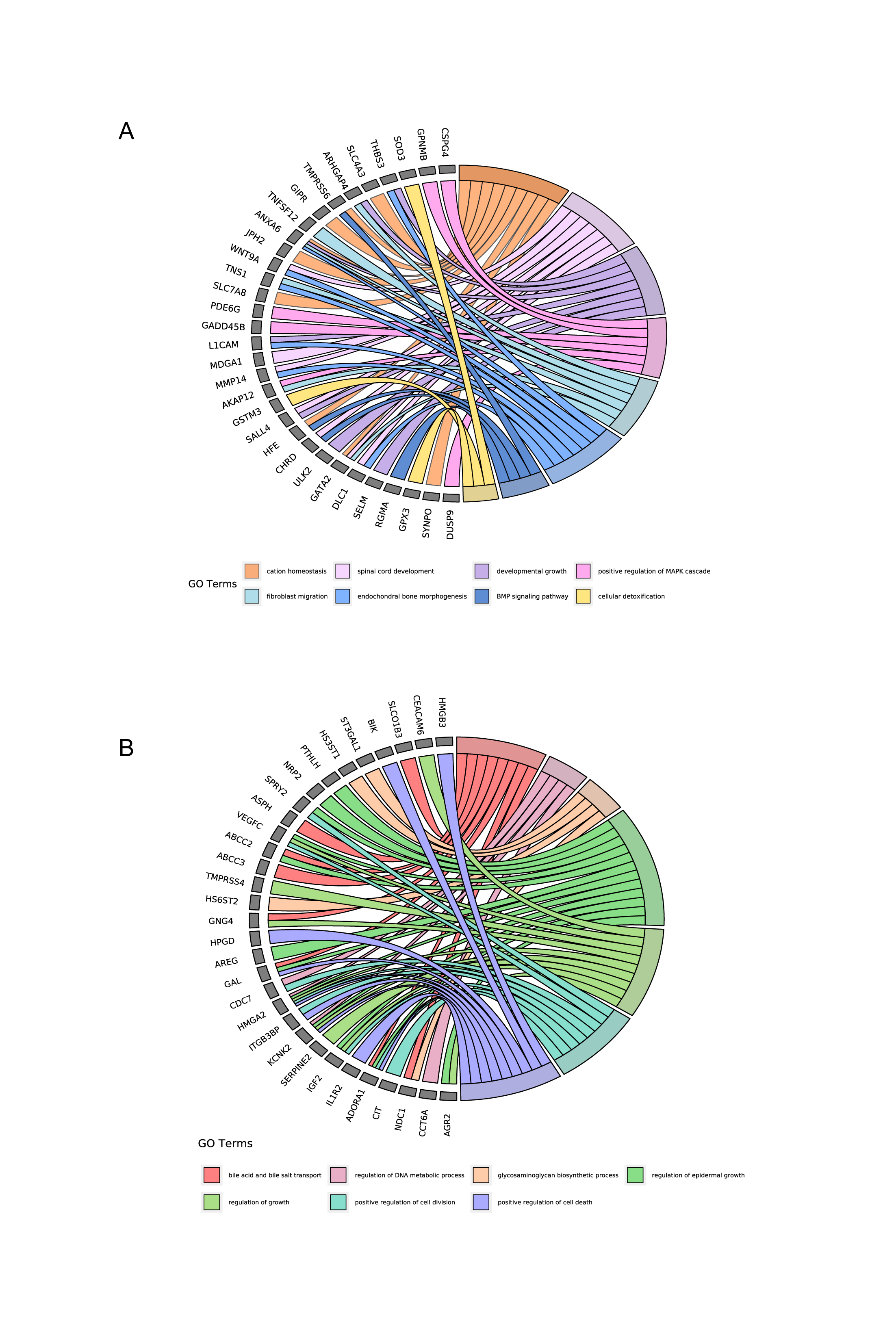

Supplement: Supplementary Figure 1 — Chord diagrams of gene ontology analysis of down-regulated (A) and upregulated (B) DEGs in DTPs. [file Image_1.tif]

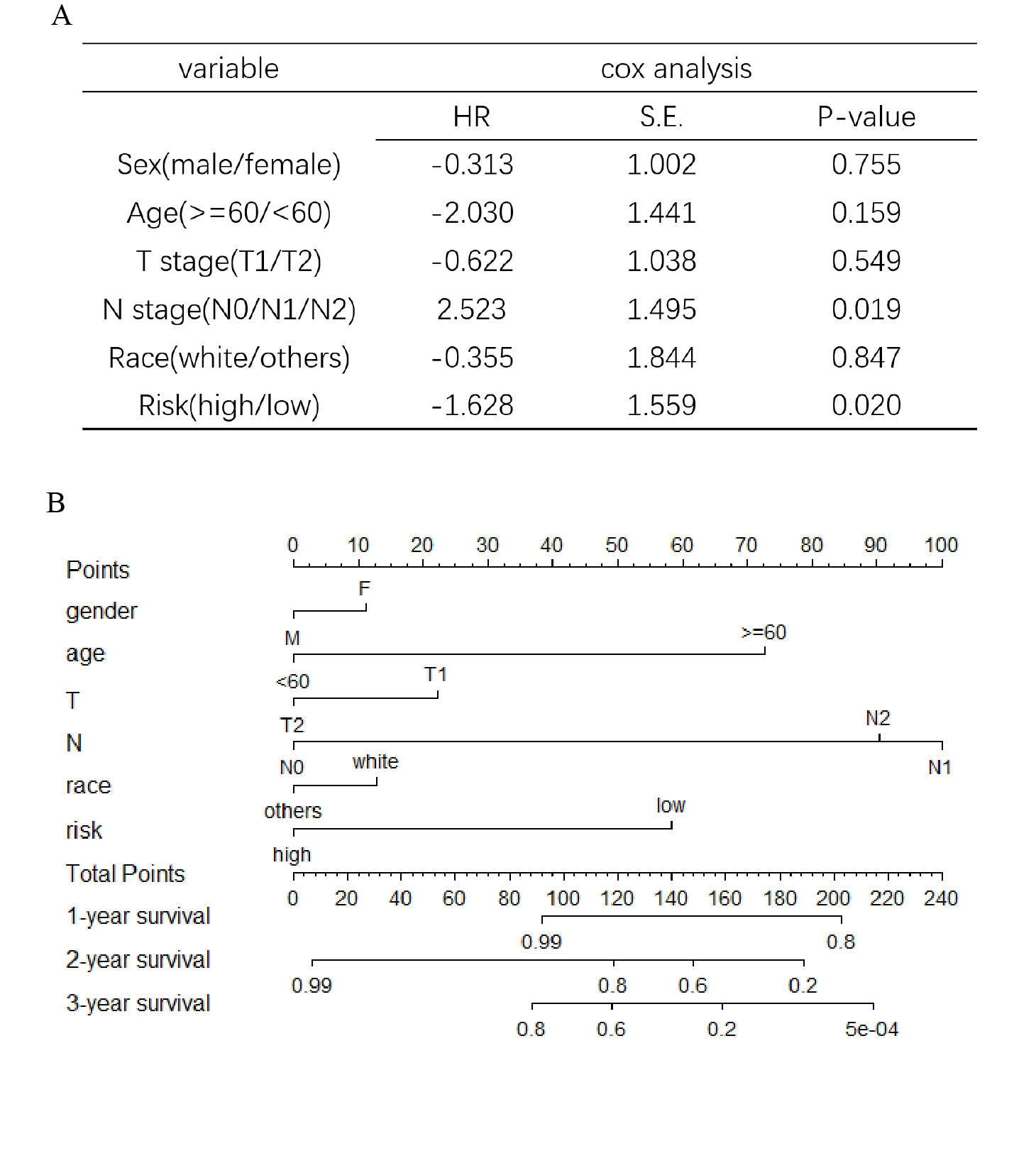

Supplement: Supplementary Figure 2 — Cox regression analysis of patient clinical information and the nomogram model for predicting abilities of NSCLC patients with 1-, 3-, and 5-year OS. [file Image_2.tif]

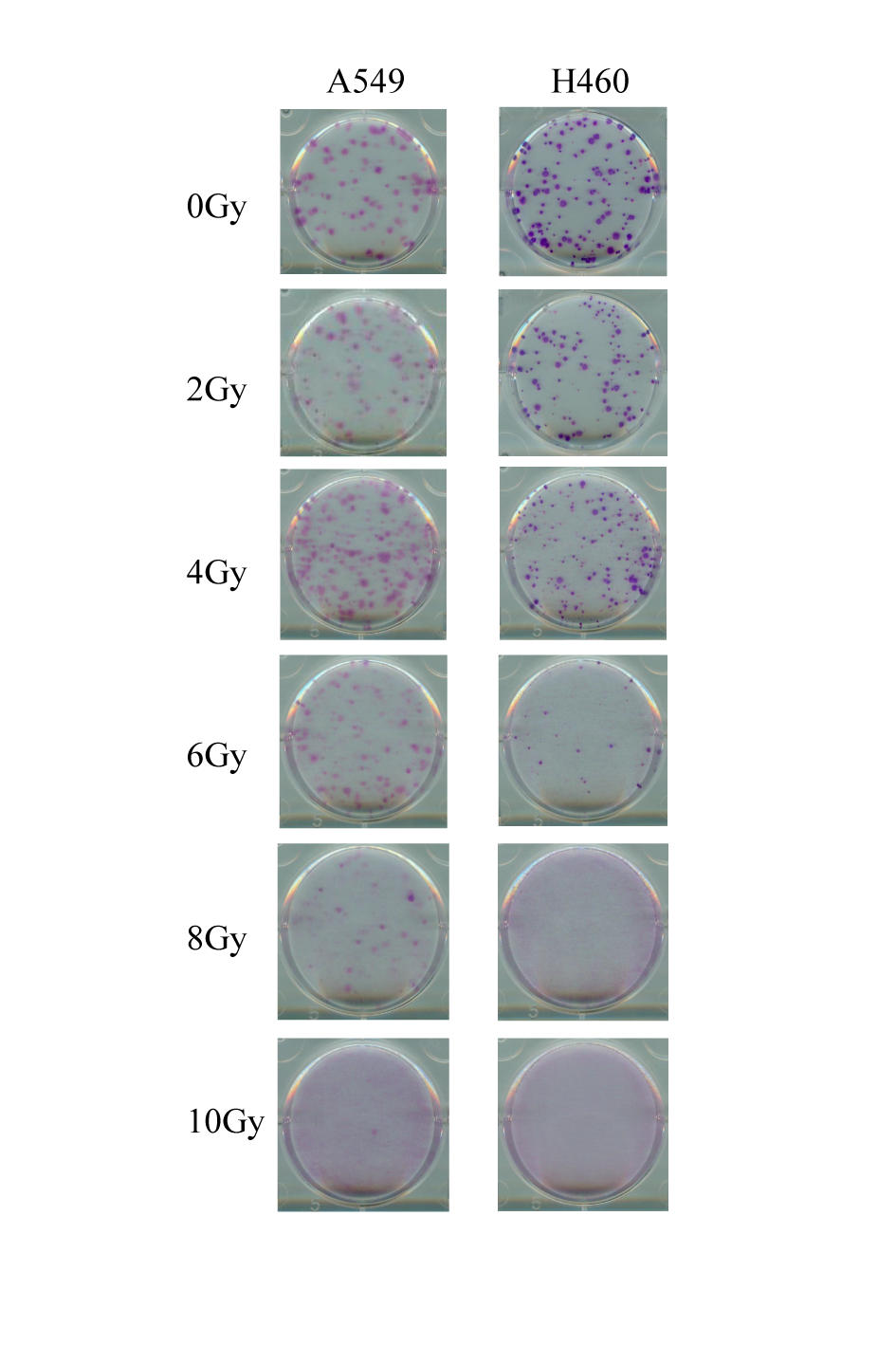

Supplement: Supplementary Figure 3 — Representative images of clongenic formation assay for comparison of A549 and H460. [file Image_3.tif]
